# Tetanus vaccine coverage in recommended and more than recommended doses among mothers in a West Cameroon health district: a cross sectional study

**DOI:** 10.12688/gatesopenres.13105.1

**Published:** 2020-04-29

**Authors:** Igor Nguemouo Nguegang, Martin Nguetsop, Linda Evans Eba Ze, Trevor Anyambod Mboh, Dominique Majoric Omokolo, Ruth Noutakdie Fossi, Etienne Guenou, Jerome Ateudjieu

**Affiliations:** 1 Faculty of Medicine and Pharmaceutical Sciences, University of Dschang, Dschang, Cameroon; 2 Meilleur Acces aux Soins de Sante (M.A. SANTE), Biyem-Assi Lac, Face Wisdome Academic complex, 33490 Yaoundé, Cameroon; 3 Department of Family Medicine, University of Manitoba, Winnipeg, Manitoba, S100, 750 Bannatyne Avenue, MB R33 0W2, Canada; 4 Elizabeth Glaser Pediatric AIDS Foundation (EGPAF), Street 1828 Ekoudou (Bastos), 35620 Yaoundé, Cameroon; 5 Division of Health Operations Research, Ministry of Public Health, Yaoundé, Cameroon

**Keywords:** Cameroon, child, mother, tetanus, vaccination, EPI

## Abstract

**Background:** Vaccination is the best way to protect newborns and mothers against tetanus. The number of doses recommended by the Expanded Program of Immunization is based on documented protective immune response. This study was conducted in 2019 in the Foumban Health District (FHD) to assess tetanus vaccine coverage among mothers for their last pregnancy and the cumulative number of vaccine doses administered to the mothers.

**Method:** This was a descriptive cross-sectional study conducted in the FHD. Mothers living in the district with at least one child younger than five years were included and were selected by random stratified cluster sampling. Trained surveyors used a face-to-face questionnaire, data extraction grid and data-tracking grid to review and collect data from antenatal care booklets, vaccination cards and the women’s own reports of immunization. The immunization coverage per vaccine dose and vaccination completeness rate were estimated.

**Results:** From 621 women visited, 602 (96.9%) responded. A total of 176/511 women (34.4%) had evidence of vaccination. For the last pregnancy, the two-dose immunization proportion was 21.7% (111/511) for documented coverage and 47.6% (243/511) for undocumented coverage. 306/570 women (53.7%) had received more than the recommended five doses necessary for lifetime protection. The recruitment, two and five doses completeness rates were 99.9% (569/570), 95.8% (546/570) and 65.3% (372/570), respectively.

**Conclusion:** More than half of mothers received more doses of tetanus toxoid vaccine than necessary in FHD. This increased the resources needed and the cost of vaccination. Health personnel should be trained and supervised to assess pregnant women’s vaccination status before planning the number of vaccine doses to be administered during pregnancy.

## Introduction

Tetanus is a non-communicable and dangerous disease caused by a toxin released from *Clostridium tetani* bacteria. In maternal tetanus, considered as tetanus during pregnancy or within six weeks of the end of pregnancy, infection occurs after abortion, miscarriage, or unhygienic delivery practices, including neonatal tetanus infection ^1^. Neonatal tetanus is defined as tetanus that occurs in the first 28 days of life. Claiming thousands of lives worldwide every year ^2, 3^, maternal and neonatal tetanus (MNT) are an important cause of maternal and neonatal mortality, almost exclusively in developing countries. In some countries, it is considered as an indicator of inequity of access to immunization and other maternal, newborn and child health services ^4^.

Maternal and neonatal tetanus prevention relies on unsafe delivery avoidance, abortion and umbilical cord care practices, and the promotion of tetanus immunization ^2, 5^. Maternal immunization with tetanus toxoid-containing vaccines (TTCVs) protects both the mother and her newborn, and is therefore a cornerstone in preventing both maternal and neonatal tetanus ^2, 3, 5– 8^. In addition to unhygienic practices during delivery, no or incomplete immunization with TTCV can be considered as a risk factor for maternal and neonatal tetanus ^2^.

By the end of 2017, tetanus immunization had a global coverage of 86% ^9^ and a Cameroon coverage of 87% ^10^. These data for vaccine coverage derived from hospital immunization registers misestimate the true proportion of protected women because of unregistered doses of tetanus toxoid ^2^. In addition, unlike a second dose of tetanus vaccine (Td2+), protection at birth (PAB) is effective when the mother has previously received protective doses for the last pregnancy, and when the mother received one dose without documentation of previous doses received. According to the immunization schedule for pregnant women and the fact that women can start vaccination against tetanus before or during their first pregnancy, the tetanus immunization coverage evaluation cannot inform on the estimated total dose of vaccine received by a woman.

The aim of this study was to assess tetanus vaccine coverage in the Foumban Health District (FHD) among mothers in terms of the number of recommended protective doses for the latest pregnancy and for the cumulative number of vaccine doses administered.

## Methods

### Ethical statement

This study was implemented following national and international regulations in research participant’s protection. All heads of household were informed and their permission obtained before meeting eligible participants. All participants were informed about the study via the information sheet and their written consent for participation and publication of the collected data was obtained before collecting their data. The information contained in their antenatal care (ANC) booklet was kept confidential and only data needed for the study were collected. We obtained ethical clearance from the National Ethical Committee for Human Health Research (N° 2019/05/20/CE/CNERSH/SP of May 6, 2019) and an administrative authorization letter from the FHD Medical Officer (N° 247/L/MINSANTE/SG/DRO/DS/FBAN of November 22, 2018).

### Study design

This was a descriptive community-based cross-sectional survey conducted among former parturient women of the FHD in 2019. A cluster randomized sampling design was used to select participants from whom trained surveyors collected the data using a face-to-face method.

### Study period

The study was conducted from July 2018 to July 2019. Recruitment and data collection were conducted from May 2019 to June 2019, following receipt of ethical approval for the study.

### Study area

The FHD is located in the west region of Cameroon, bordered by four other health districts of the same region and by the North West Region in the North. It has a surface area of 1,734 km². The FHD is made up of 22 health areas with a total population of 235,828 inhabitants in 2018 ^11^. This district was chosen because it gives an opportunity to pilot the exploration of access to immunization among pregnant women in an environment with strong religious and socio-cultural influences on access to care.

### Study population

The target population was women of childbearing age with at least one child aged 0–59 months living in FHD for at least three months. Those absent from selected households for two visits in a week were not included.

### Sample size

The sample size was estimated at 620, using a confidence interval of 95% and precision of 5% and assuming the immunization coverage in the Cameroon general population to be 73.9% from a previous demographic health survey (DHS 2011) ^12^, a design effect due to cluster sampling of two and response rate of 94.4% from DHS 2011.

### Sampling procedure

The estimated sample size was assigned to 10 health areas (HA) selected from the 22 HA of the FHD by a stratified random sampling process. At the HA level, 62 quarters were selected using a systematic sampling method. The number of clusters per HA was proportional to the total population of the HA and 10 mothers were included per quarter. The listing of all localities was done in alphabetical order with the cumulative population frequency. The sample interval in each HA was determined by dividing the total population of the area by the allocated number of clusters. In each village/quarter, one direction or street was randomly selected from the centre. We included the first household on the right and another one after, skipping two households. We further chose the participant in the selected households. If there were two or more mothers in a household, we randomly chose one, and if there were no eligible woman, the next household was included in the study.

### Data collection instrument

 The data collection tools were developed by the study team and pre-tested among 14 women in the community for fluency, acceptance by participants and precision. Following pre-testing, the questionnaire was divided into a questionnaire, a data extraction grid and a data tracking grid and some questions were adapted or removed to make questions more pertinent and reduce the time taken to administer the questionnaire (from ~30 minutes to ~10 minutes). The questionnaire (see *Extended data*) ^13^ was used to collect data on socio-demographic variables, knowledge of ANC and the tetanus vaccine. One data extraction grid and one data-tracking grid were also used; the first when ANC booklet or immunization card were available and the second when they were not (see *Extended data*) ^14, 15^.

### Data collection procedure

Trained surveyors conducted a face-to-face interview at the participant’s home to collect data from eligible mothers. Surveyors were male and female community health workers with at least three years of experience. Before beginning, the aim of the study was explained in the information sheet and written informed consent was obtained. Interviews were 5–10 minutes in duration. Data collected included: age; education level; marital status; profession; parity; knowledge of tetanus vaccine; availability of ANC booklet and immunization card; and doses of tetanus toxoid vaccine received since their first pregnancy, during their last pregnancy and during their last three pregnancies, with earlier pregnancies not considered to reduce the possibility of memory bias. The information sources were recall and evidence of vaccination (Td cards and ANC booklets).

### Bias

There was a high probability of recall bias as data were collected using a questionnaire. We cannot be certain of the respondent statements, as some may not have a good memory of vaccination from many years ago or some could for some reason hide or deform some answers. The research team could not overcome this weakness as it cannot be avoided when data are collected using questionnaires. Nevertheless, some key variables were collected from vaccination cards filled out by health personnel and we believe these have less risk of being affected by information bias.

### Data analysis

We assessed the survey coverage and response rate per cluster to mitigate the risk of selection bias. Data were analyzed by estimating the proportion of mothers with each dose of tetanus vaccine received, the proportion of documented immunization coverage, undocumented immunization coverage and stratification by education level. Attempting to infer results to the general population, proportions were estimated with 95% confidence intervals. Data were entered and analyzed using Epi Info version 7.2.2.6 ^16^. Missing data were considered missing and were not replaced; however, the common denominator for the groups of variables was kept unchanged.

## Results

### Socio-demographic characteristics

Out of 621 participants reached, 602 responded, giving a response rate of 96.9% (CI 95%: 95.6-98.3). The mean age of participants was 28.4±8 years. The coverage rate of the 62 clusters was 100% and the response rate varied from 94.3% to 100%. Out of 602 participants, 483 were Muslim (80.2%, CI 95%: 77.1-83.4) and 319 were housewives (52.9%, CI 95%: 49.0-56.9). Secondary education was the most represented education level among participants. Table 1 shows the distribution of participants by socio-demographic characteristics ^17^.

**Table 1.** Distribution of participants according to socio-demographic characteristics.

Socio-demographic characteristics ( N= 602)	Frequency (n)	Proportion (%)	CI 95%	
**Age (years)**				
≤19	50	8.4	6.4	10.8
20–24	164	27.4	23.9	31.1
25–35	270	45.1	41.1	49.1
>35	115	19.2	16.2	22.6
**Education level**				
Illiterate	30	4.9	3.2	6.7
Primary	239	39.7	35.8	43.6
Secondary	321	53.3	49.3	57.3
Higher	6	1.0	0.2	1.8
**Main profession**				
Civil servant	35	5.8	3.9	7.7
Farmer	32	5.3	3.5	7.1
Trader	112	18.6	15.5	21.7
Housewife	319	52.9	49.0	56.9
Other	92	15.3	12.4	18.2
**Marital status**				
Single	69	11.5	8.9	14.0
Married	477	79.2	76.0	82.5
Divorced	33	5.5	3.7	7.3
Widow	20	3.3	1.9	4.8
**Age of first pregnancy** **(years)**				
<=19	410	71.4	67.6	74.9
20–24	137	23.9	20.6	27.5
25–35	27	4.7	3.3	6.8
>t35	0	0.0	0.0	0.0

### Tetanus immunization coverage for last pregnancy

In our study, 176/511 (34.4%, CI 95%: 30.3-38.6) women had ANC booklets or immunization cards (Td card). 111/511 (21.7%, CI 95%: 18.1-25.3) participants had documented immunization coverage of two or more tetanus doses (Td2+) and 243/511 (47.6%, CI 95%: 43.2-51.9) had undocumented immunization coverage. The high proportion of unprotected births 102/280 (36.4%, CI 95%: 30.8-42.1) was among those education to secondary education level. Table 2 presents the distribution of tetanus vaccination coverage at the last pregnancy.

**Table 2.** Distribution of mother tetanus vaccine coverage at the last pregnancy in the Foumban Health District.

Tetanus dose received during ANC	Education level [ n (%) ]				Total (n=511)
	Illiterate (n=23)	Primary (n=203)	Secondary (n=280)	Higher (n=5)	
**Immunization status from Td card or** **ANC booklet**					
Less than two vaccination doses	0 (0.0)	17 (8.4)	48 (17.1)	0 (0.0)	65 (12.7)
At least two vaccination doses	2 (8.7)	46 (22.7)	62 (22.1)	1 (20.0)	111 (21.7)
*Subtotal*	2 (8.7)	63 (31.0)	110 (39..3)	1(20.0)	176 (34.4)
**Undocumented Immunization status**					
Less than two vaccination doses	5 (21.7)	32 (15.8)	54 (19.3)	1 (20.0)	92 (18.0)
At least two vaccination doses	16 (69.6)	108 (53.2)	116 (41.4)	3 (60.0)	243 (47.6)
*Subtotal*	21 (91.3)	140 (68.9)	170 (60.7)	4 (80.0)	335 (65.6)
**Documented and undocumented** **Vaccination status**					
Less than two vaccination doses	5 (21.7)	49 (24.1)	102 (36.4)	1 (20.0)	157 (30.7)
At least two vaccination doses	18 (78.3)	154 (75.9)	178 (63.6)	4 (80.0)	354 (69.3)

Td card, tetanus vaccine immunization card; ANC, antenatal care.

### Cumulative and completeness vaccination coverage

The recruitment, two and five doses completeness rates were 569/570 (99.9%, CI 95%: 99.5-100), 546/570 (95.8%, CI 95%: 94.2-97.5) and 372/570 (65.3%, CI 95%: 61.4-69.2), respectively. Among former parturient women, 306/570 (53.7%, CI 95%: 49.6-57.8) had received more than five tetanus vaccine doses ( Table 3). The number of doses increased with the number of pregnancies. Table 3 and Table 4 present the distribution of tetanus immunization coverage per dose by education level and the variation of distribution of estimated cumulate tetanus vaccine dose received with the number of pregnancies.

**Table 3.** Distribution of tetanus immunization coverage per dose classified by education level in the Foumban Health District.

Estimated cumulative Td vaccine dose received	Education level [n (%)]				Total (n=570)
	Illiterate (n=25)	Primary (n=230)	Secondary (n=309)	Higher (n=6)	
0	0 (0.0)	0 (0.0)	1 (0.32)	0 (0.0)	1 (0.2)
At least 1	25 (100.0)	230 (99.9)	308 (99.7)	6 (100.0)	569 (99.9)
At least 2	25 (100.0)	226 (98.3)	289 (93.5)	6 (100.0)	546 (95.8)
At least 5	21 (84.0)	169 (73.5)	179 (54.9)	3 (50.0)	372 (65.3)
More than 5	17 (68.0)	149 (64.8)	140 (45.3)	0 (0.0)	306 (53.7)

**Table 4.** Variation of distribution of estimated cumulative tetanus vaccine doses received with the number of pregnancies in the Foumban Health District.

Estimated cumulative vaccine dose received	Number of pregnancies [ n (%) ]				Total (n=576)
	1 (n=89)	2 (n=104)	3 (n=105)	More than 3 (n=278)	
0	0 (0.0)	0 (0.0)	1 (0.9)	0 (0.0)	1 (0.2)
5	89 (100.0)	95 (91.4)	42 (40.0)	39 (14.0)	265 (46.0)
10	0 (0.0)	9 (8.7)	62 (59.1)	141 (50.7)	212 (36.8)
11 – 15	0 (0.0)	0 (0.0)	0 (0.0)	71 (25.5)	71 (12.3)
>15	0 (0.0)	0 (0.0)	0 (0.0)	27 (9.7)	27 (4.7)

## Discussion

The documented vaccination coverage of at least two doses of tetanus vaccine at the end of the previous pregnancy was 21.7% and increases to 69.3% when the mother's statements about their vaccination status (undocumented vaccination coverage) are taken into account. Taking into account the total number of tetanus vaccine doses received during all pregnancies by each woman, the first contact, two and five doses completeness rate was 99.9%, 95.8% and 65.3%, respectively. Over half (53.3%) of participants had received more than five tetanus vaccine doses.

To protect the child from tetanus, it is recommended that a minimum of two doses of this vaccine be administered to each woman before the end of pregnancy ^9, 18^. Routinely, the estimation of two-dose vaccination coverage is done by calculating the proportion of women who received the vaccine in the antenatal consultation follow-up register ^19^. However, since not all women attend antenatal consultations, the community-based survey is more suitable for this estimation. The best source of data for this estimation is the vaccination card or ANC booklet. The present study shows a significant difference between documented and declared vaccination coverage and highlights the need to identify and respond to the reasons for this difference and to decide on the data sources to be used to monitor vaccination coverage ^20^. In the present paper, we considered and discussed vaccination coverage, taking into account the vaccination card and the mother's declarations. This approach is supported by the argument that the survey was done less than five years after the last pregnancy, with good chances that mothers remember their vaccination status.

The two-dose immunization coverage from immunization cards and declarations indicates that three out of 10 babies are born unprotected against tetanus. The fact that a significant proportion of newborns are unprotected against tetanus is concerning for our national elimination status. This could be explained by limits in the supply of vaccination ^21^, the limited access of pregnant women to organized vaccination sessions or the fact that the estimate of vaccination coverage fails to include vaccine doses administered during previous pregnancies. Taking into account the fact that in most health facilities, ANC and vaccination against tetanus are integrated ^22^, noted in the ANC booklet and given to the pregnant woman, and that these women change their booklet from one pregnancy to the next, we opted in this study to estimate the cumulative vaccination coverage of the mother taking into account all vaccine doses administered in previous pregnancies.

The tetanus immunization schedule during pregnancy in Cameroon is based on the WHO recommendations ^18^. This schedule recommends administering at least two doses at least four weeks apart, the last of which must be administered at least two weeks before delivery; the three booster doses administered with an interval of at least six, 12 and 24 months after the second dose, which provide five, 10 and all childbearing age years protection, respectively ^10, 23^.

Our results showed that 65.3% of mothers had completed five doses of the tetanus vaccine and that 53.7% had received more than the five maximum recommended doses for lifetime protection ^24^. Without evidence of an existent study, this is the first study that assesses the cumulative administered vaccine to this targeted population. The present study did not investigate reasons for this situation. From our experience in observing and supervising vaccination sessions, it can result from some weaknesses in planning, delivering and monitoring tetanus toxoid vaccination in pregnancy. These may include the fact that: 1) in practice, the denominator recommended by the Expanded Program on Immunization to monitor the coverage of tetanus toxoid vaccine is the estimated number of pregnant women (assuming all of them have not been previously vaccinated) ^19^; 2) the evaluation of vaccination status is not conducted before planning the number of vaccine doses to be administered in each pregnancy and; 3) ANC booklets also serve as vaccination cards and are renewed with each pregnancy. No study has shown any danger administering more doses of tetanus vaccines than necessary to pregnant women. However, there is no doubt that this situation increases the cost of vaccination, as shown in previous studies ^21, 25^, in terms of the number of vaccine doses to be purchased and human resources needed to organize vaccination sessions and also leads to underestimates of the performance of the Expanded Program on Immunization.

### Limitations and strengths of the study

The estimated coverage of vaccination during the last pregnancy and cumulative number of doses from all pregnancies was expected to be based on data collected from the Td card or ANC booklet; however, only a third of the participants had evidence of vaccination. We had to collect this data using a tracking tool to assess the vaccination status of women that had no evidence of vaccination. Using two data sources to estimate this coverage can lead to information bias that may question the validity of our results. This is a difficulty encountered in almost all of the vaccination coverage surveys that we tried to solve by using a vaccine status-tracking grid. The limitations of the current study have lead our team to launch two other studies: a study to document and respond to reasons for the unavailability of evidence of vaccination and a study to test tools that allow with certain reliability to determine the vaccination status of women who have no evidence of vaccination.

## Conclusion

The present study indicates that more than half of the participants received more doses of vaccines than recommended for adequate protection against tetanus of the mother and the child during the mother's childbearing years. This increases the need for resources and the cost of vaccination. This study also showed that the vaccine coverage and the level of protection of this vaccine in pregnant women are underestimated if estimates do not take into account the cumulative number of doses of vaccine received by the mother before and during previous pregnancies. To improve the situation, we recommend training health personnel to assess the vaccination status before planning the number of doses of vaccines to be administered to a pregnant woman. Moreover, the same document should be used for all antenatal consultations across pregnancies to allow a better assessment of the number of vaccine doses already administered. Scientists should identify and respond to the reasons for the unavailability of evidence of vaccination and test vaccine status tracking tools in women who have no source of verification of vaccine status.

## Data availability

### Underlying data

Figshare: Tetanus vaccine coverage in recommended and more than recommended doses among mothers in a West Cameroon Health District: MetaData. https://doi.org/10.6084/m9.figshare.11803248.v1 ^17^

### Extended data

Figshare: Questionnaire. https://doi.org/10.6084/m9.figshare.11828625.v1 ^13^

Figshare: Data extraction grid. https://doi.org/10.6084/m9.figshare.11828643.v1 ^14^

Figshare: Data tracking grid. https://doi.org/10.6084/m9.figshare.11828697.v1 ^15^

Data are available under the terms of the Creative Commons Zero "No rights reserved" data waiver (CC0 1.0 Public domain dedication).
