# Supplementary material for: Tetanus vaccine coverage in recommended and more than recommended doses among mothers in a West Cameroon health district: a cross sectional study
Source: Gates Open Res. 2021 Mar 10;4:46. Originally published 2020 Apr 29. [Version 2] doi: 10.12688/gatesopenres.13105.2 (PMC11813171; doi:10.12688/gatesopenres.13105.2)
Supplement: Peer review history

# Reviewer report: Reviewer response for version 2

**DOI:** 10.21956/gatesopenres.14440.r30781

**Published:** 2024-12-27

**Authors:** Karnaboopathy Ranganathan

**Affiliations:** 1 Department of Community Medicine, Shri Sathya Sai Medical College and Research Institute, Kanchipuram, Tamil Nadu, India

Comments against Abstract and other part of the article is given below:

 1. Study design mentioned in the title " a cross sectional study" but it's mentioned in the abstract and methods are "a descriptive cross-sectional study".

 2. Method of sampling mentioned in the abstract is "random stratified cluster sampling" Mention the proper sampling method.

 3. The demographic health survey response rate 94.4% mentioned in the sample size calculation, but in the result part mentioned in the response rate is 96.9%. why this variation?

 4. In the result part mentioned the frequency with corresponding percentage and 95% CI, what is the use of 95% CI in frequency distribution without any statistical significant?

Is the work clearly and accurately presented and does it cite the current literature?

Yes

If applicable, is the statistical analysis and its interpretation appropriate?

Partly

Are all the source data underlying the results available to ensure full reproducibility?

Partly

Is the study design appropriate and is the work technically sound?

Yes

Are the conclusions drawn adequately supported by the results?

Partly

Are sufficient details of methods and analysis provided to allow replication by others?

Yes

Reviewer Expertise:

1. Statistics

I confirm that I have read this submission and believe that I have an appropriate level of expertise to confirm that it is of an acceptable scientific standard.

# Reviewer report: Reviewer response for version 2

**DOI:** 10.21956/gatesopenres.14440.r38969

**Published:** 2024-12-24

**Authors:** Florence A. Kanu

**Affiliations:** 1 Centers for Disease Control and Prevention, Atlanta, Georgia, USA

Thank you for the opportunity to review this study. Overall, the aim of this study was to assess tetanus vaccine coverage in FHD among mothers, specifically evaluating the number of doses received during last pregnancy and the cumulative number of vaccine doses administered. Besides being a descriptive study on maternal tetanus vaccine uptake, it is not clear what this aim attempts to add to the scientific literature on this topic. The abstract mentions that FHD was “the seat of an epidemic despite the satisfactory administrative immunization coverage” but this point is not expanded upon anywhere else in the manuscript to aid in justifying the purpose of this study. The authors conclude that more than half of mothers received more Td doses than recommended and that this increased the resources needed and cost of vaccination; however, resource allocation and cost to administer the vaccine was not evaluated as part of this study to justify such a conclusion. It is recommended that this manuscript be reframed to explain why this study was warranted and present results that best support their conclusions and answers the study aim.

 According to WHO guidance on sustaining MNTE (Page 44), tetanus vaccination status should be verified by card or history during the first ANC visit. If card and/or history confirm that the pregnant woman is fully protected against tetanus (i.e. has received 6 TTCV doses in childhood/adolescence, or 5 doses if first vaccinated after 1 year of age/during adolescence/adulthood, including during previous pregnancies), then no further vaccination is needed. In this case, it is very important to record the pregnant woman as “fully vaccinated with TTCV” on ANC card and in the immunization register, even though she did not receive any TTCV doses in the current pregnancy. This point should be included in the discussion section as it highlights a potential opportunity to rectify the experience of mothers receiving more than the recommended number of Td doses.

 Below are my comments by section for consideration:

 Introduction

- The authors state that Cameroon’s tetanus immunization coverage was 87% at the end of 2017 but it is not clear if this is Td2+ coverage or PAB coverage. In review of the Td2+ and PAB data available on WHO’s website, Td2+ coverage was 69% and 63% in 2017 and 2018, respectively and PAB coverage was 85% in both 2017 and 2018. However, I suggest that the authors clarify which coverage estimate is presented here and cite an appropriate data source.
- The final paragraph of the introduction could be expanded to not only mention the aim of the study but to justify why this study is warranted.

Results

- Authors do not explain how the results go from 602 respondents to a denominator of 511. The 2 ^nd^ paragraph states that 176/511 reportedly had ANC booklets or immunization cards and 111/511 had documented Td2+ immunization coverage. Does this mean the other 400 did not have documentation? It may be better to say 176/511 had ANC booklets or immunization cards and of those 111/176 had Td2+ documentation.
- The authors go on to mention 243/511 had “undocumented immunization coverage” but does not define this term. Does this mean that 243 participants recalled receiving Td vaccine but did not have a record of such vaccination in their ANC booklet or immunization card? As asked above, does this 243 include part of the 400 that did not have documentation? What about the rest?
- Revise the first sentence under “cumulative and completeness vaccination coverage” as it is unclear the way it is currently written.
- Would suggest include results on cost of vaccination and resource allocation (if measured) given the conclusions specifically mention that providing more than the recommended number of doses led to increases in cost and resources.

Discussion

- The 2 ^nd^ paragraph in the discussion mentions “the present study shows a significant difference between documented and declared vaccination coverage” but the results do not reference a test of significance. Please include first in the results before mentioning in the discussion.
- When discussing the proportion of babies unprotected against tetanus at birth, the authors mention that reasons for this could be “limits in the supply of vaccination, limited access of pregnant women to organize vaccination sessions or the fact that the estimate of vaccination coverage fails to include vaccine doses administered during previous pregnancies”. Were these asked about in the questionnaire? If so, these data should be presented in the results first before offered as a potential justification for the 3 out of 10 infants unprotected at birth against tetanus.
- “53.7% had received more than five maximum recommended doses for lifetime protection” it would be helpful if the average and range were presented for the group of women who received more than the recommended amount in the results.
- In the second to last paragraph, the authors write “However, there is no doubt that this situation increases the cost of vaccination” but data on cost were not presented in the results. It is suggested that the authors provide data (if assessed) on cost to justify this conclusion.
- In the limitation section, the authors mention that they tried to resolve the issue of 2 data sources by using a vaccine status-tracking grid. It would be helpful if the authors could expand on how a vaccine status tracking grid aided in resolving this issue.

Conclusion

- “This increases the need for resources and the cost of vaccination” Not sure this conclusion can be made given that this was not formally assessed as part of this study.
- With the final sentence in the conclusion section, the authors note that they are doing exactly what they suggest “scientists should” do (they mention this in the limitation section) so not sure if this final sentence is needed in the conclusion section.

Tables

- Table 1: would seem more appropriate to include “none” as an education level rather than “illiterate”. This suggested revision applies to all tables that include the word “illiterate”
- Table 2: The subtotal % for the section ‘immunization status from Td card or ANC booklet’ for secondary education includes an extra decimal point (written as 39..3).
- Table 2 & 3: it is not clear if the proportions in the cells are row or column percents as they do not add up to 100 when reviewed by row or column. Please revise so it reflects either a row or column percent.

Is the work clearly and accurately presented and does it cite the current literature?

No

If applicable, is the statistical analysis and its interpretation appropriate?

Partly

Are all the source data underlying the results available to ensure full reproducibility?

Partly

Is the study design appropriate and is the work technically sound?

Partly

Are the conclusions drawn adequately supported by the results?

No

Are sufficient details of methods and analysis provided to allow replication by others?

Yes

Reviewer Expertise:

Immunization; tetanus vaccination; maternal and neonatal tetanus elimination

I confirm that I have read this submission and believe that I have an appropriate level of expertise to state that I do not consider it to be of an acceptable scientific standard, for reasons outlined above.

# Reviewer report: Reviewer response for version 2

**DOI:** 10.21956/gatesopenres.14440.r30778

**Published:** 2021-07-12

**Authors:** M. Carolina Danovaro-Holliday

**Affiliations:** 1 Strategic Information Group, Expanded Program on Immunization (EPI), Department of Immunization, Vaccines and Biologicals (IVB), World Health Organization (WHO), Geneva, Switzerland

The authors report the results of a survey (not using probability sampling) to assess vaccination with a tetanus toxoid containing vaccine (TTCV) conducted in a health district in Cameroon in 2019. While the main results will likely stand, i.e., excess TTCV vaccination for some women and not enough vaccination for others, more clarifications are needed to fully evaluate the findings.

**My main suggestions are:**

- Consider properly reviewing the existing literature to put the findings into context, notably the most updated WHO Recommendations synthesized in the 2017 Tetanus vaccine Position paper: https://www.who.int/teams/immunization-vaccines-and-biologicals/policies/position-papers/tetanus . The main WHO reference used https://www.who.int/reproductivehealth/publications/maternal_perinatal_health/immunization_tetanus.pdf is outdated (2002).
- Provide the reader with information about the TTCV recommendations in Cameroon. What is the recommended schedule? How many booster doses are recommended? Which vaccine is used, TT or Td (and since when, if Td)?
- Describe better the survey methods used. The study reported used a method of quota sampling no longer endorsed by the World Health Organization (WHO), see: https://www.who.int/teams/immunization-vaccines-and-biologicals/immunization-analysis-and-insights/global-monitoring/immunization-coverage/survey-methods .
  - Why did the authors chose this methodology rather than probability sampling?
  - Why were women who gave birth, but with stillbirths and infants who died, excluded? (against current WHO recommendations – see Survey Manual in link provided above).
  - Provide more information about the survey implementing teams and analysis. Where can the reader find the database and analytical code?
  - Mention that this method assumes self-weighting and that the uncertainty estimates (95% Confidence intervals in this case), thus, are difficult to interpret. Also unclear how the sampling design was handled in the analysis
- With all these, the limitations section must be expanded to also discuss selection bias (non-probability sampling, unclear revisit procedures, who selected households and documentation of HHs approached vs. surveyed) and ascertainment bias linked not only to long recall period, limited availability of written records, but also to who asks and how questions are asked to ascertain vaccination status.
- Tell us how the findings of the survey results are going to be used to change policy and/or practices in Cameroon.

**Comments by section:**

Abstract:

- Consider adding “and safe birth and umbilical cord care practices” to this sentence “Vaccination is the best way to protect newborns and mothers against tetanus”.
- Please revise this sentence “During this decade, the Foumban Health District (FHD) was the seat of an epidemic despite the satisfactory administrative immunization coverage”.
  - Tetanus does not result in epidemics.
  - What is meant by “satisfactory administrative immunization coverage”? Infant coverage with three doses of diphtheria-tetanus-pertussis (DTP3)? TTCV coverage among…women of reproductive age? Pregnant women? Provide a level, >80%, 90%, something else?
- Tell us about occurrence of maternal and neonatal tetanus in FHD, the MNT still a public health problem?
- In the methods subsection, indicate the recall period, i.e., mother of children born aged 0-59 months at the time of the survey.
- I suggest explaining the recommendation briefly in the abstract, as two doses of TTCV during each pregnancy is *not* a blanket recommendation. It only applies in certain circumstances. In fact, the 2017 WHO Position Paper on Tetanus Vaccines states: “Women who have documented evidence that they received all of the doses needed for full protection, i.e. either 6 TTCV doses in childhood or 5 doses if first vaccinated during adolescence/adulthood, should not be further vaccinated during pregnancy in order to avoid the risk of increased local reactions.”
- It is unclear to me why the proportions presented use 511 (the number of women with some evidence of vaccination) as the denominator. We read that the number of eligible women included was 602, and this should be the denominator to calculate proportion vaccinated, as done in most household surveys. Thus, what happened to the other 91 women [602-511]?
- I also don’t understand this statement “The recruitment, two and five doses completeness rates were 99.9% (569/570), 95.8% (546/570) and 65.3% (372/570), respectively” – why 570 as denominator? What does it mean by “the recruitment?

Introduction:

- Consider a more comprehensive review of existing recommendations, notably the latest WHO position paper.
- Provide background information about the Foumban Health District (FHD), tetanus there, and tetanus recommendations in Cameroon.
- Td seems to be used as synonym with TT. Please clarify. Since when is Td used in Cameroon?
- Explain what is Protection at Birth (PAB) vis-à-vis coverage with a TTCV.
- On the later, “By the end of 2017, tetanus immunization had a global coverage of 86%...” is not supported by the references provided (link is broken), plus WHO only estimated PAB (until 2017) not coverage.
- I cannot access reference 10 either to check this part “… and a Cameroon coverage of 87%” – please revise and update link.

Methods:

- How was written informed consent handled for illiterate women?
- Were the participants informed if they needed a TTCV dose?
- Tell us about data management, cleaning and analysis other than done with EpiInfo. Paper-based data collection? Single data entry?

Results:

- Table 1. It is difficult to interpret the 95%CI included in table 1. Also, as totals don’t add to 602, you may want to add a row of missing in each category.
- It may be better to provide mean and dispersion for continues variables in the text.
- This section is missing how we go from 602 to denominators of 511 and to 570 for different indicators. Why?

Discussion:

- The first paragraph just repeats data already provided. Better if the authors summarize in their words the key findings.
- This statement: “To protect the child from tetanus, it is recommended that a minimum of two doses of this vaccine be administered to each woman before the end of pregnancy” must be revised in light of current tetanus vaccine recommendations. Reference 9 is inaccessible using the link provided and reference 18 is outdated.
- Reference 20, about validity of recall in surveys is okay, but there are two more recent and complete reviews on this topic: Modi *et al.* (2018 ^1^) and Dansereau *et al.* (2020 ^2^).
- Revise section about WHO recommendations, as citation 18 is outdated as mentioned before.
- A Tunisia paper (ref 19) is used to talk about WHO recommendations. This needs to be supported by a primary document (though the Tunisia experience can be kept as a good explanation on how it was used over twenty years ago).
- This statement is *not *accurate “No study has shown any danger administering more doses of tetanus vaccines than necessary to pregnant women.” In fact, the 2017 WHO Position Paper highlights the increase in adverse reactions following additional doses “The intensity and frequency of both local and systemic reactions tend to increase with an increasing number of previous vaccinations. For instance, redness of the injection site was reported in 12.2%, 16.2%, and 19.4% of vaccinees following the first, second and third doses of DTaP in the primary series, and 31.4% following receipt of any dose including booster doses. There is also a risk of administering excess TT when pre-existing tetanus antibody levels are already high, which can result in increased local reaction rates.” – You can look at the accompanying documentation of the Position Paper to look for primary research so you can cite papers about increases in adverse events, notably local reactions.
- Expand the limitation section to account for potential biases related to the sampling procedures used.

References:

- There are several broken links that must be updated.

Data sharing:

- Where can we access the analytical code used?

Is the work clearly and accurately presented and does it cite the current literature?

No

If applicable, is the statistical analysis and its interpretation appropriate?

Partly

Are all the source data underlying the results available to ensure full reproducibility?

Yes

Is the study design appropriate and is the work technically sound?

Partly

Are the conclusions drawn adequately supported by the results?

Partly

Are sufficient details of methods and analysis provided to allow replication by others?

Partly

Reviewer Expertise:

Immunization

I confirm that I have read this submission and believe that I have an appropriate level of expertise to state that I do not consider it to be of an acceptable scientific standard, for reasons outlined above.

## References

[1] : Caregiver recall in childhood vaccination surveys: Systematic review of recall quality and use in low- and middle-income settings. *Vaccine* .2018;36(29) : 10.1016/j.vaccine.2018.05.089 4161-4170 10.1016/j.vaccine.2018.05.089 29885771

[2] : A systematic review of the agreement of recall, home-based records, facility records, BCG scar, and serology for ascertaining vaccination status in low and middle-income countries. *Gates Open Research* .2020;3: 10.12688/gatesopenres.12916.2 10.12688/gatesopenres.12916.2 PMC711094132270134

# Reviewer report: Reviewer response for version 2

**DOI:** 10.21956/gatesopenres.14440.r30777

**Published:** 2021-07-06

**Authors:** Maria Rosário Fraga Oliveira Martins

**Affiliations:** 1 Global Health and Tropical Medicine, Universidade Nova de Lisboa, Lisboa, Portugal

The objective of this study was to **assess tetanus vaccine coverage among mothers for their last pregnancy** and the cumulative number of vaccine doses administered to the mothers. It was conducted during 2019 in Foumban Health District (FHD). FHD is located in the west region of Cameroon, bordered by four other health districts of the same region and by the North West Region in the North. It is made up of 22 health areas with a total population of 235,828 inhabitants in 2018.

**Methods**

 To attain the objective, the authors conducted a descriptive community-based cross-sectional survey conducted among former parturient women. A cluster randomized sampling design was used to select participants.

 The sample size was computed using a confidence interval of 95% and precision of 5% and assuming the immunization coverage in the Cameroon general population to be 73.9% from a previous demographic health survey (DHS 2011) ^. ^A design effect due to cluster sampling of two was considered together with a response rate of 94.4% (from DHS 2011).

 The target population was women of childbearing age with at least one child aged 0–59 months living in FHD for at least three months. Those absent from selected households for two visits in a week were not included.

 The estimated sample size was 620 women.

 In Data analysis the authors referred that in attempting to infer results to the general population, proportions were estimated with 95% confidence intervals.

**Comment 1**

 The authors want to infer which results?

 In the main objective and in sample size calculation it is mentioned that the primary outcome is tetanus vaccine coverage among mothers for their last pregnancy. And only for this outcome, it will be possible to infer something to the population. Must be clarified.

**Results**

 Tables 1, 2 and 3

**Comment 2**

 The number of women with Higher education is so low (n=6) that I wonder if the result has real meaning; also there are no women with age of first pregnancy >35 years; does it make sense to have a line of zeros?

**Comment 3**

 I can’t see why the CI95% are presented together with socio-demographic characteristics. It should be presented together with the main outcome, but the sample was not chosen to be representative of the socioeconomic level. I am not sure of the added value of this information

**Discussion**

**Comment 4**

 In my opinion, if the authors want to infer something to the population we need to know more about the target population. Some statistics at the population level would be very useful to compare with the results from this study. For example: what are the sociodemographic characteristics of women of childbearing age with at least one child aged 0–59 months living in FHD? (namely age, level of education, profession and marital status).  It’s important to compare the sample used in this study with the population characteristics to evaluate the extent to which it is representative or not. If not there will be a possible bias, namely in disaggregated data that should also be mentioned.

Is the work clearly and accurately presented and does it cite the current literature?

Yes

If applicable, is the statistical analysis and its interpretation appropriate?

Partly

Are all the source data underlying the results available to ensure full reproducibility?

Yes

Is the study design appropriate and is the work technically sound?

Yes

Are the conclusions drawn adequately supported by the results?

Yes

Are sufficient details of methods and analysis provided to allow replication by others?

Partly

Reviewer Expertise:

Statistics, Public Health

I confirm that I have read this submission and believe that I have an appropriate level of expertise to confirm that it is of an acceptable scientific standard, however I have significant reservations, as outlined above.

# Reviewer report: Reviewer response for version 1

**DOI:** 10.21956/gatesopenres.14270.r29118

**Published:** 2020-09-14

**Authors:** Ghose Bishwajt

**Affiliations:** 1 Faculty of Social Sciences, School of International Development and Global Studies, University of Ottawa, Ottawa, ON, Canada

- Foumban Health District is not a standard term, it needs more clarification.
- Year of survey needs to be mentioned in the abstract.
- The definition of outcome variable also needs to be mentioned in the abstract.
- The first 2 paragraphs in the introduction are very general in description and should be replaced by something more substantial: contextual factors, what progress has been made so far, what challenges lie ahead, what should be done etc.
- The following study was not cited: Chiabi *et al.* (2017 ^1^).
- What the present study is contributing to the literature should also be mentioned.

Is the work clearly and accurately presented and does it cite the current literature?

Yes

If applicable, is the statistical analysis and its interpretation appropriate?

Partly

Are all the source data underlying the results available to ensure full reproducibility?

Yes

Is the study design appropriate and is the work technically sound?

Partly

Are the conclusions drawn adequately supported by the results?

Yes

Are sufficient details of methods and analysis provided to allow replication by others?

No

Reviewer Expertise:

Maternal and child health

I confirm that I have read this submission and believe that I have an appropriate level of expertise to confirm that it is of an acceptable scientific standard, however I have significant reservations, as outlined above.

## References

[1] : Vaccination of infants aged 0 to 11 months at the Yaounde Gynaeco-obstetric and pediatric hospital in Cameroon: how complete and how timely?. *BMC Pediatr* .2017;17(1) : 10.1186/s12887-017-0954-1 206 10.1186/s12887-017-0954-1 29258463 PMC5735527

# Response

**Published:** 2020-11-05

**Authors:** Igor NGUEGANG

Thank you Sir Ghose Bishwajt for your critical reading and comments on this article; We will consider them when correcting.

 Best, 

# Reviewer report: Reviewer response for version 1

**DOI:** 10.21956/gatesopenres.14270.r28822

**Published:** 2020-06-11

**Authors:** Jayani Pathirana

**Affiliations:** 1 Medical Research Council, Respiratory and Meningeal Pathogens Research Unit, Faculty of Health Science, University of the Witwatersrand, Johannesburg, South Africa

This is a descriptive cross sectional study from Cameroon to assess receipt of the appropriate doses of tetanus vaccines by mothers for protective immunity against tetanus. The authors interviewed women of child bearing age with at least one child and reviewed ANC and immunisation records and if unavailable relied on maternal recall to determine tetanus vaccination history.

 The key finding in this study was that over 50% of women had received greater than 5 doses of tetanus vaccine, the required number for long-term protection against tetanus. The authors also identified 36.4% of births to be unprotected from tetanus.

 This is an important study which highlights vaccine wastage as women received more than the required doses of tetanus vaccine. The authors were limited by unavailability of documentation on maternal vaccination status with reliance on maternal recall. There is however a disconnect between the background provided, the aim of the study and the results described. Specific comments are provided below. 

**Abstract:**

**Background:**

1. The background would benefit from including the justification for the study (why was this study undertaken?).
2. The authors stated: "the number of doses recommended by the Expanded Program of Immunization...". Doses of which vaccine? Please specify the vaccine being studied. 

**Introduction:**

1. "infection occurs after abortion, miscarriage...". This implies that tetanus always occurs following these events. Authors should clarify, providing references, that the main cause of tetanus infection is from unhygienic delivery practices which may occur during childbirths or miscarriage/abortion (spontaneous or induced) and may also result in neonatal tetanus.
2. Suggest that authors include standard definition for PAB with a reference. 
3. Have the authors reviewed the literature for similar studies where more than the recommended  tetanus doses are administered to pregnant women and the reasons for this? If there are no other studies, clarify this in the methods. 

**Methods:**

**Ethical statement:**

1. How were heads of household contacted and informed about the study? 

**Study design: **

1. Suggest that authors clarify that method was "face-to-face" interviews. 
2. Was a woman with a live birth an inclusion criteria, or were women with stillbirths and miscarriages included.

**Data collection instrument:**

1. The authors should clarify whether it is standard practice for women to have home based ANC records or whether ANC cards are usually kept at health care facilities. Authors should also clarify whether tetanus immunization is recorded in ANC records or an independent immunization card or both.

**Data collection procedure:**

1. Suggest the authors clarify whether maternal recall is equivalent to undocumented immunization. 

**Data analysis:**

1. The authors should clarify the importance of stratifying by education level. 
2. There is no mention of the source of funding. 

**Results:**

1. It is recommended to not begin sentences with numeric e.g. 111/115 etc.

**Tetanus immunization coverage for last pregnancy:**

1. The authors mention "unprotected at birth". Was PAB measured and if so how? This would need to be clarified in the methods. Maternal TT/Td coverage is not equivalent to PAB. 

**Cumulative and completeness vaccination coverage:**

1. Expanding on the details of Table 4 would be useful as the results need to be standing alone from the tables. 

Is the work clearly and accurately presented and does it cite the current literature?

Partly

If applicable, is the statistical analysis and its interpretation appropriate?

Yes

Are all the source data underlying the results available to ensure full reproducibility?

Yes

Is the study design appropriate and is the work technically sound?

Yes

Are the conclusions drawn adequately supported by the results?

Yes

Are sufficient details of methods and analysis provided to allow replication by others?

Partly

Reviewer Expertise:

Vaccinology, epidemiology, public health

I confirm that I have read this submission and believe that I have an appropriate level of expertise to confirm that it is of an acceptable scientific standard, however I have significant reservations, as outlined above.

# Response

**Published:** 2020-11-05

**Authors:** Igor NGUEGANG

Thank you Sir Jayani Pathirana for the time you have taken to review the paper. Your suggestion will be taken into account.

 In response to your concerns, the study was conducted to access the Td vaccine community coverage because despite the good national coverage published, this region was victim of many epidemics situation of certain diseases under surveillance.

 During the implementation, data were collected:

     - using a face-to-face method from women of childbearing age with at least one child, after obtaining the verbal authorization of their husband (according to the cultural reason of the study area) and the written consent of the women concerned.

     - from the antenatal care booklets or vaccination cards (depending of their availability at the health facilities) because, in our context, theses evidences of immunization are given to the women who are responsible for keeping them.

     - from maternal recall in case of absence of the previous document (undocumented immunization). 

 Cordially,
